# Supplementary material for: Regional Topological Aberrances of White Matter- and Gray Matter-Based Functional Networks for Attention Processing May Foster Traumatic Brain Injury-Related Attention Deficits in Adults
Source: Brain Sci. 2021 Dec 24;12(1):16. doi: 10.3390/brainsci12010016 (PMC8774280; doi:10.3390/brainsci12010016)
Supplement: Supplementary file 1 [file brainsci-12-00016-s001.zip › Supplementary_Materials.pdf]

Table S1. Nodes for White Matter Functional Brain Network Construction

Anatomical Regions	Abbreviations	Local Maxima in MNI coordinates of power spectrum		
		x	y	z
Genu_of_corpus_callosum	GCC	-2	24	10
Body_of_corpus_callosum	BCC	2	-28	22
Splenium_of_corpus_callosum	SCC	0	-38	12
Fornix	FX	0	-8	18
Cerebral_peduncle_L	CP_L	-16	-12	-6
Anterior_limb_of_internal_capsule_R	ALIC_R	18	2	12
Anterior_limb_of_internal_capsule_L	ALIC_L	-22	8	16
Posterior_limb_of_internal_capsule_R	PLIC_R	26	-20	10
Posterior_limb_of_internal_capsule_L	PLIC_L	-18	-16	-2
Retrolenticular_part_of_internal_capsule_R	RLIC_R	30	-26	4
Retrolenticular_part_of_internal_capsule_L	RLIC_L	-34	-36	6
Anterior_corona_radiata_R	ACR_R	24	30	8
Anterior_corona_radiata_L	ACR_L	-22	28	12
Superior_corona_radiata_R	SCR_R	18	6	28
Superior_corona_radiata_L	SCR_L	-18	-16	40
Posterior_corona_radiata_R	PCR_R	26	-48	26
Posterior_corona_radiata_L	PCR_L	-26	-40	26
Posterior_thalamic_radiation_R	PTR_R	36	-56	4
Posterior_thalamic_radiation_L	PTR_L	-34	-54	2
Sagittal_stratum_R	SS_R	40	-28	-10
Sagittal_stratum_L	SS_L	-40	-28	-10
External_capsule_R	EC_R	34	-6	-10
External_capsule_L	EC_L	-32	6	-6
Cingulum_(cingulate_gyrus)_R	CgC_R	8	-26	34
Cingulum_(cingulate_gyrus)_L	CgC_L	-8	-26	34
Cingulum_(hippocampus)_R	CgH_R	20	-40	-2
Cingulum_(hippocampus)_L	CgH_L	-18	-40	-4
Fornix cres or stria terminalis_R	FX/ST_R	28	-24	-4
Fornix cres or stria terminalis _L	FX/ST_L	-28	-28	-2
Anterior_Superior_longitudinal_fasciculus_R	ASLF_R	34	4	24
Middle_Superior_longitudinal_fasciculus_R	MSLF_R	34	-22	34
Posterior_Superior_longitudinal_fasciculus_R	PSLF_R	40	-46	8
Anterior_Superior_longitudinal_fasciculus_L	ASLF_L	-34	6	22
Middle_Superior_longitudinal_fasciculus_L	MSLF_L	-36	-34	32
Posterior_Superior_longitudinal_fasciculus_L	PSLF_L	-32	-44	30
Superior_fronto-occipital_fasciculus_R	SFO_R	20	0	24
Superior_fronto-occipital_fasciculus_L	SFO_L	-22	6	22
Uncinate_fasciculus_R	UNC_R	34	-2	-14
Uncinate_fasciculus_L	UNC_L	-36	-2	-18
Tapetum_R	TAP_R	30	-46	16
Tapetum_L	TAP_L	-26	-46	18

Table S2. Nodes for Gray Matter Functional Brain Network Construction

Anatomical Regions	Abbreviations	Local Maxima in MNI coordinates of brain activation		
		x	y	z
L. Basal ganglia, globus pallidus	BG_L_6_2	-22	-2	4
L. Basal ganglia, ventromedial putamen	BG_L_6_4	-23	7	-4
L. Basal ganglia, dorsal caudate	BG_L_6_5	-14	2	16
L. Basal ganglia, dorsolateral putamen	BG_L_6_6	-28	-5	2
R. Basal ganglia, globus pallidus	BG_R_6_2	22	-2	3
R. Basal ganglia, ventromedial putamen	BG_R_6_4	22	8	-1
R. Basal ganglia, dorsolateral putamen	BG_R_6_6	29	-3	1
L. Cingulate gyrus, caudodorsal	CG_L_7_5	-5	7	37
R. Cingulate gyrus, pregenual	CG_R_7_3	5	28	27
L. Fusiform gyrus, medioventral	FuG_L_3_2	-31	-64	-14
L. Fusiform gyrus, lateroventral	FuG_L_3_3	-42	-51	-17
R. Fusiform gyrus, medioventral	FuG_R_3_2	31	-62	-14
R. Fusiform gyrus, lateroventral	FuG_R_3_3	43	-49	-19
L. Inferior frontal gyrus, dorsal	IFG_L_6_1	-46	13	24
L. Inferior frontal gyrus, opercular	IFG_L_6_5	-39	23	4
L. Inferior frontal gyrus, ventral	IFG_L_6_6	-52	13	6
R. Inferior frontal gyrus, dorsal	IFG_R_6_1	45	16	25
R. Inferior frontal sulcus	IFG_R_6_2	48	35	13
R. Inferior frontal gyrus, caudal	IFG_R_6_3	54	24	12
R. Inferior frontal gyrus, opercular	IFG_R_6_5	42	22	3
R. Inferior frontal gyrus, ventral	IFG_R_6_6	54	14	11
L. Dorsal agranular insula	INS_L_6_3	-34	18	1
L. Dorsal granular insula	INS_L_6_5	-38	-8	8
L. Dorsal dysgranular insula	INS_L_6_6	-38	5	5
R. Dorsal agranular insula	INS_R_6_3	36	18	1
R. Dorsal dysgranular insula	INS_R_6_6	38	5	5
L. Inferior parietal lobule, rostrodorsal	IPL_L_6_2	-38	-61	46
L. Inferior Parietal Lobule, rostrodorsal	IPL_L_6_3	-51	-33	42
L. Inferior parietal lobule, caudal	IPL_L_6_4	-56	-49	38
L. Inferior parietal lobule, rostroventral	IPL_L_6_6	-53	-31	23
R. Inferior parietal lobule, rostrodorsal	IPL_R_6_2	39	-65	44
R. Inferior parietal lobule, rostrodorsal	IPL_R_6_3	47	-35	45
R. Inferior parietal lobule, caudal	IPL_R_6_4	57	-44	38
R. Inferior parietal lobule, rostroventral	IPL_R_6_5	53	-54	25
L. Inferior temporal gyrus, extreme lateroventral	ITG_L_7_2	-51	-57	-15
L. Inferior temporal gyrus, ventrolateral	ITG_L_7_5	-55	-60	-6
L. Inferior temporal gyrus, caudolateral	ITG_L_7_6	-59	-42	-16
R. Inferior temporal gyrus, extreme lateroventral	ITG_R_7_2	53	-52	-18
R. Inferior temporal gyrus, ventrolateral	ITG_R_7_5	54	-57	-8
R. Inferior temporal gyrus, caudolateral	ITG_R_7_6	61	-40	-17
L. Middle occipital gyrus	LOcC_L_4_1	-31	-89	11
L. lateral occipital cortex	LOcC_L_4_2	-46	-74	3

Table S2 (Continued). Nodes for Gray Matter Functional Brain Network Construction

Anatomical Regions	Abbreviations	Local Maxima in MNI coordinates of brain activation		
		x	y	z
L. Occipital polar cortex	LOcC_L_4_3	-18	-99	2
L. Inferior occipital gyrus	LOcC_L_4_4	-30	-88	-12
L. Middle frontal gyrus, dorsal	MFG_L_7_1	-27	43	31
L. Inferior frontal junction	MFG_L_7_2	-42	13	36
L. Middle frontal gyrus	MFG_L_7_3	-28	56	12
L. Middle frontal gyrus, ventral	MFG_L_7_4	-41	41	16
L. Middle frontal gyrus, ventrolateral	MFG_L_7_5	-33	23	45
L. Middle frontal gyrus, ventrolateral	MFG_L_7_6	-32	4	55
R. Middle frontal gyrus, dorsal	MFG_R_7_1	30	37	36
R. Inferior frontal junction	MFG_R_7_2	42	11	39
R. Middle frontal gyrus	MFG_R_7_3	28	55	17
R. Middle frontal gyrus, ventral	MFG_R_7_4	42	44	14
R. Middle frontal gyrus, ventrolateral	MFG_R_7_5	42	27	39
R. Middle frontal gyrus, ventrolateral	MFG_R_7_6	34	8	54
R. Middle frontal gyrus, lateral	MFG_R_7_7	25	61	-4
L. Middle temporal gyrus, caudal	MTG_L_4_1	-65	-30	-12
L. Anterior superior temporal sulcus	MTG_L_4_4	-58	-20	-9
R. Middle temporal gyrus, caudal	MTG_R_4_1	65	-29	-13
R. Middle temporal gyrus, dorsolateral	MTG_R_4_3	60	-53	3
R. Anterior superior temporal sulcus	MTG_R_4_4	58	-16	-10
L. Orbital gyrus, lateral	OrG_L_6_3	-23	38	-18
L. Orbital gyrus, lateral	OrG_L_6_6	-41	32	-9
R. Orbital gyrus, orbital	OrG_R_6_2	40	39	-14
R. Orbital gyrus, lateral	OrG_R_6_3	23	36	-18
R. Orbital gyrus, lateral	OrG_R_6_6	42	31	-9
L. Postcentral gyrus (upper limb, head and face region)	PoG_L_4_1	-50	-16	43
L. Postcentral gyrus (tongue and larynx region)	PoG_L_4_2	-56	-14	16
L. Postcentral gyrus	PoG_L_4_3	-46	-30	50
L. Postcentral gyrus (trunk region)	PoG_L_4_4	-21	-35	68
R. Postcentral gyrus	PoG_R_4_3	48	-24	48
L. Precentral gyrus (head and face region)	PrG_L_6_1	-49	-8	39
L. Precentral gyrus, caudal dorsolateral	PrG_L_6_2	-32	-9	58
L. Precentral gyrus (upper limb region)	PrG_L_6_3	-26	-25	63
L. Precentral gyrus (trunk region)	PrG_L_6_4	-13	-20	73
L. Precentral gyrus (tongue and larynx region)	PrG_L_6_5	-52	0	8
L. Precentral gyrus, caudal ventrolateral	PrG_L_6_6	-49	5	30
R. Precentral gyrus, caudal dorsolateral	PrG_R_6_2	33	-7	57
R. Precentral gyrus (tongue and larynx region)	PrG_R_6_5	54	4	9
R. Precentral gyrus, caudal ventrolateral	PrG_R_6_6	51	7	30
L. Rostroposterior superior temporal sulcus	pSTS_L_2_1	-54	-40	4
L. Caudoposterior superior temporal sulcus	pSTS_L_2_2	-52	-50	11

Table S2 (Continued). Nodes for Gray Matter Functional Brain Network Construction

Anatomical Regions	Abbreviations	Local Maxima in MNI coordinates of brain activation		
		x	y	z
R. Rostroposterior superior temporal sulcus	pSTS_R_2_1	53	-37	3
R. Caudoposterior superior temporal sulcus	pSTS_R_2_2	57	-40	12
R. Lateral superior occipital gyrus	LOcC_R_2_2	29	-75	36
R. Middle occipital gyrus	LOcC_R_4_1	34	-86	11
R. Lateral occipital cortex	LOcC_R_4_2	48	-70	-1
R. Occipital polar cortex	LOcC_R_4_3	22	-97	4
R. Inferior occipital gyrus	LOcC_R_4_4	32	-85	-12
L. Superior frontal gyrus, medial	SFG_L_7_1	-5	15	54
L. Superior frontal gyrus, dorsolateral	SFG_L_7_4	-18	-1	65
L. Superior frontal gyrus, medial	SFG_L_7_5	-6	-5	58
L. Superior frontal gyrus, medial	SFG_L_7_6	-5	36	38
R. Superior frontal gyrus, medial	SFG_R_7_1	7	16	54
R. Superior frontal gyrus, dorsolateral	SFG_R_7_4	20	4	64
R. Superior frontal gyrus, medial	SFG_R_7_5	7	-4	60
R. Superior frontal gyrus, medial	SFG_R_7_6	6	38	35
L. Superior parietal lobule, lateral	SPL_L_5_3	-33	-47	50
L. Superior parietal lobule, postcentral	SPL_L_5_4	-22	-47	65
L. Superior parietal lobule, intraparietal	SPL_L_5_5	-27	-59	54
R. Superior parietal lobule, lateral	SPL_R_5_3	35	-42	54
R. Superior parietal lobule, intraparietal	SPL_R_5_5	31	-54	53
L. Superior temporal gyrus	STG_L_6_2	-54	-32	12
L. Superior temporal gyrus, caudal	STG_L_6_4	-62	-33	7
L. Thalamus, medial pre-frontal	Tha_L_8_1	-7	-12	5
L. Thalamus, pre-motor	Tha_L_8_2	-18	-13	3
L. Thalamus, sensory	Tha_L_8_3	-18	-23	4
L. Thalamus, posterior parietal	Tha_L_8_5	-16	-24	6
L. Thalamus, caudal temporal	Tha_L_8_7	-12	-22	13
L. Thalamus, lateral pre-frontal	Tha_L_8_8	-11	-14	2
R. Thalamus, medial pre-frontal	Tha_R_8_1	7	-11	6
R. Thalamus, pre-motor	Tha_R_8_2	12	-14	1
R. Thalamus, lateral pre-frontal	Tha_R_8_8	13	-16	7

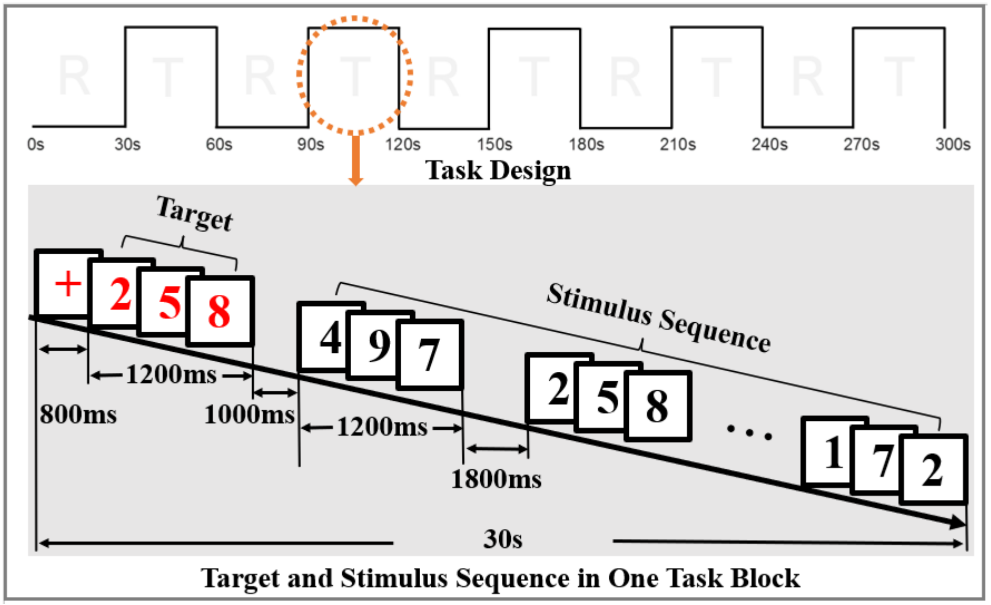

Figure S1: Functional MRI experimental task design. (ms: millisecond; s: second)
